# A retrospective analysis of 62,571 cases of perioperative adverse events in thoracic surgery at a tertiary care teaching hospital in a developing country

**DOI:** 10.1186/s13019-019-0921-z

**Published:** 2019-05-31

**Authors:** Qiongzhen Li, Xiaofeng Zhang, Meiying Xu, Jingxiang Wu

**Affiliations:** Department of Anesthesiology of Shanghai Chest Hospital, Shanghai Jiao Tong University, Shanghai, 200030 China

**Keywords:** Thoracic surgery, Perioperative, Adverse events

## Abstract

**Objectives:**

Despite a long history of concerns regarding patient safety during clinical care, some patients undergoing thoracic surgery continue to experience adverse events (AEs). AEs are a major significant source of perioperative morbidity and mortality following thoracic surgery. This study analysed the causes, treatment and prognosis of perioperative AEs to provide a reference for further surgical safety.

**Methods:**

The authors collected a total of 62,571 thoracic surgery anaesthesia records via the Anaesthesia Information Management System (AIMS) from 14 August 2006 to 14 August 2017 and obtained 150 cases of perioperative serious AEs from the “adverse events registration” subsystem. The related hospitalization data of the 150 patients were analysed, including anaesthesia, recovery room time, ICU records and follow-up outcomes. The causes of these AEs were classified as follows: events related to the patients’ pathogenic conditions(P); surgery-related factors(S); anaesthesia-related factors(A); and interactions between pathogenic, surgical and anaesthesia factors (P&S&A). We then analysed the main clinical manifestations, causes and treatment of these events.

**Results:**

The overall rate of perioperative AEs in thoracic surgery (*n* = 62,571) was 0.2%. Of these, 10.7% were.

caused by P and 23.3% by A; neither cause led to patient death. S and P&S&A accounted for 55.3 and 10.7% of AEs, respectively; together, they accounted for 66%. Twelve patients with postoperative AEs caused by S or P&S&A died within 3 days (8% of 150 cases). A total of 33%(50/150) of patients experienced sudden cardiac arrest (SCA) and recovered successfully. Surgical massive haemorrhage (22%, 33/150) was reported as a predominant mortality-related outcome in this group, and 8 of the 12 deaths were caused by massive haemorrhage.

**Conclusions:**

The rate of perioperative AEs after thoracic surgery was 0.2%. AEs must be identified and treated immediately. An important factor in anaesthesia-related events was respiratory management. Two major clinical manifestations of surgery-related events were cardiac arrest and massive haemorrhage. Cardiac arrest was the major factor contributing to AEs, but its adverse consequences could be avoided with timely discovery and proper treatment. Massive haemorrhage is a significant cause of mortality that can be prevented with a surgeon’s early diagnosis and appropriate interventions.

## Background

Anaesthesia-related mortality rates and complications have declined considerably [1–3]. This decrease has been attributed to a variety of safety improvements, including new anaesthetic drugs, anaesthetic techniques, monitoring techniques and the development and widespread adoption of practice guidelines [1–3]. However, patient safety issues remain a problem worthy of sustained attention. Some surgical patients may still suffer from unexpected events during hospitalization, especially during cardiac, thoracic, vascular, gastrointestinal, paediatric and orthopaedic surgery [4–6].

Thoracic surgery is an independent risk factor for perioperative complications, especially in patients with comorbidities before surgery [7, 8]. The adverse events reporting system is a measure to improve patient safety by recording and analysing the causes, patterns and characteristics of events [9, 10]. Surgeons can obtain feedback and draw from these failures to identify aspects of their processes or operations to adopt, modify or avoid [9, 11, 12]. Therefore, in the past 11 years, an electronic adverse events reporting system has been incorporated into the anaesthesia information management system at Shanghai Chest Hospital. Anaesthesia is a special medical process during surgery, and perioperative adverse events often involve many factors, including the patient’s pre-existing illness, surgical trauma, stimulation and deprivation resulting from the operation, anaesthesia, and the operation itself. Thoracic surgery, which involves the thoracic cavity, may affect the heart, lungs and autonomic nervous system. Therefore, it is challenging for anaesthesiologists to rapidly diagnose and properly treat perioperative adverse events.

In this study, we analysed the causes, treatment and prognosis of 150 out of 62,571 perioperative adverse events during thoracic surgery over an 11-year period to provide a reference for improving surgical safety.

## Methods

### Patients and data collection

The study protocol was approved by the Institutional Review Board of Shanghai Jiaotong University Shanghai Chest Hospital (KS-2017-18) for operations that require general anaesthesia. For this study, we collected a total of 62,571 thoracic surgery anaesthesia records via the Anaesthesia Information Management System (AIMS) from August 142,006 to August 142,017. The patients underwent lung surgery (*n* = 49,732), oesophageal surgery (*n* = 4975), mediastinal surgery (*n* = 6991), tracheal surgery (*n* = 581), and other surgery (*n* = 292). We obtained 150 cases of perioperative serious adverse events from the “adverse events registration” system .

Perioperative adverse events were defined as unfavourable intraoperative incidents and postoperative complications. An adverse event was defined as “an event affecting patient safety that occurred during the perioperative period and needed special medical treatment for relief” [13]. We focused on the preoperative period (from the preoperative anaesthesia visit to 48 h after anaesthesia). For each operation, trained nurse data reviewers prospectively collected data in a standardized manner [14]. The collected variables included postoperative complications over a 2-day period. The Department of Anaesthesia at our tertiary care hospital conducts regular monthly morbidity and mortality review meetings at which any adverse events leading to major or intermediate patient morbidity and/or mortality within the 48-h perioperative period are reviewed, discussed and recorded.

The related hospitalization data of the 150 patients, including anaesthesia, recovery room time, ICU records and follow-up outcomes, were retrospectively analysed. The causes of these adverse events were classified as follows: events related to the patients’ pathogenic conditions(P); surgery-related factors(S); anaesthesia-related factors(A); and interactions among these three factors(P&S&A). We then further analysed the main clinical manifestations, causes and treatments of these events.

### Statistical analysis

The data are expressed as the mean ± standard deviation. Descriptive statistics are reported as frequencies and percentages. Statistical analyses were performed using SPSS version 22 (SPSS, Chicago, IL). Descriptive statistics were performed on all categorical and continuous data elements using the χ2 test. A *P* value< 0.05 was considered significant.

## Results

We collected a total of 62,571 thoracic surgery cases. The majority (49,732 cases, 79.5%) involved lung surgery, including 48.6% thoracoscopic surgeries; there were also 4975 cases (8.0%) of oesophageal surgery, 6991 cases of mediastinal surgery, including 35.2% mediastinoscopy surgeries, 581 cases of tracheal surgery, and 292 cases of other surgery.

The total rate of adverse events was 0.2%. The rate among cases of tracheal surgery was 0.7%, which was higher than the rates for lung surgery (0.2%, *p* < 0.05), mediastinal surgery (0.3%, p < 0.05) and oesophageal surgery (0.4%, p < 0.05). Cardiac arrest(33%, 50/150) was the major contributing factor to adverse events, but all cardiac arrest cases were successfully resuscitated. Surgical massive haemorrhage(22%, 33/150) was a significant cause of mortality in this group.

The classification of the 150 cases of adverse events according to the different causes is shown in Table 1. Eleven percent were caused by P, and 23% were caused by A; neither cause resulted in patient deaths. Events caused by S and P&S&A accounted for 55 and 11%, respectively, and together accounted for 66% of the adverse events. Of all cases, 12 postoperative patients with adverse events caused by S or P&S&A died within 3 days (8% of 150 cases). Ten of these 12 died from massive haemorrhage. Regarding the other 2 cases, one died from the intraoperative falling of a left atrial mural tumour; the other recovered successfully from an intraoperative cardiac arrest but died of a recurrent cardiac arrest 3 days later (Table 1).

**Table 1:** Distribution and Ratio of Adverse Events in Different Categories

Categories	n	n(%)	Death	Deaths(%)
P	16	11	0	0
A	35	23	0	0
S	83	55	9	75
P&S&A	16	11	3	25
Total	150	100	12	100

P = events related to the patients’ pathogenic conditions, *S* surgery-related events, *A* anaesthesia -related events, *P&S&A* events related to interaction of the three causative factors

The adverse events caused by P included adverse drug events (4 cases), a transfusion reaction (1 case), autoimmune disease factors (6 cases), intraoperative bronchial haemorrhage(2 cases), and arrhythmias caused by excessive hypoglycaemia (3 cases; Table 2).

**Table 2:** Details of events related to the patients’ pathogenic conditions

Operation	Clinical manifestation	Causes	Treatment
Right lower lobectomy	Left bronchus bleeding after endotracheal intubation with a F32 double lumen tube	Upper left lobe bronchiectasis	Fibreoptic bronchoscopy, operation postponed until 12 days later
Video-assisted mediastinoscopic lymphnode biopsy	Arrhythmia (frequent premature ventricular with different QRS wave directions and tachycardia-bradycardia syndrome)	Hypoglycaemia 2.3 mmol/L	Arrhythmia relieved after the infusion of glucose
Right lower lobectomy	Limb twitching and spasms, atrial ectopic beat, and hyperpnoea and hypertension after ondansetron injection	Rare adverse drug reaction	Oxygen inhalation, midazolam 2 mg, amiodarone 150 mg
Pneumonectomy of right lung cancer	Hypotension and tachycardia after the administration of hydroxyethyl starch	Hydroxyethyl starch allergy	Stopped hydroxyethyl starch infusion, dilation with crystal solution, low-dose epinephrine
Pneumonectomy of destroyed right lung	Hypotension, chemosis and high airway pressure after blood transfusion	Transfusion reaction	Stopped blood transfusion and added infusion of low-dose epinephrine

The 35 cases of adverse events caused by A (Table 3) were classified into the following three categories: (1) airway problems (24 cases), including unexpected difficult airway (13 cases), severe hypoxia during awake fibreoptic bronchoscope intubation in trachea tumour patients with complete airway obstruction (2 cases), bronchial spasm of the laryngeal mask airway for anaesthesia (4 cases), and severe airway problems after extubation-induced significant hypoxia (5 cases); (2) cycling-related problems(3 cases), including one case who was breathing spontaneously via an endotracheal tube and suffered cardiac arrest (ventricular fibrillation) during anaesthesia recovery, cycled stably after 40 min of cardiopulmonary resuscitation (including continuous cardiac massage, epinephrine and other drugs, and clicking defibrillation) and woke after 8 h, and another case of a lung resection patient who exhibited a mediastinal swing during transition and presented a sharp drop in blood pressure; (3) other problems, such as drug misbranding (1 case); (4) puncture haematoma (3 cases); and (5) teeth falling into the trachea or mouth (3 cases). The anaesthesia-related problems were handled and resolved well without sequelae or complications.

**Table 3:** Details of anaesthesia-related events

Categories	n	Clinical manifestation	Treatment
Unexpected difficult airway	10	Difficult laryngeal exposure with Macintosh and Glidescope laryngoscope; pressure oxygen mask was not effective	Fibreoptic bronchoscope-guided intubation (*n* = 7) Shikani seeing optical stylet-guided intubation (*n* = 1) Tracheotomy (*n* = 2)
	3	Laryngeal oedema and dyspnoea	Intubation with an F5.5 tracheal tube +high-frequency ventilation
Airway occlusion	2	Severe dyspnoea caused by complete tracheal occlusion by a tracheal tumour during fibreoptic bronchoscope-guided awake intubation in a patient scheduled for tracheal surgery	Emergency cardiopulmonary bypass (CPB) Operation performed on CPB
Bronchial spasm	4	SPO_2_ declined sharply with high airway pressure	Corticosteroid+endotracheal epinephrine
Airway problems after extubation	3	Upper respiratory tract obstruction after extubation in patient with laryngocarcinoma operation history or radical resection of tongue carcinoma	Pressure oxygen mask and oropharyngeal airway
	2	Dyspnea after double lumen tube extubation Laryngeal edema	Re-intubation and corticosteroids
Haemodynamic instability	3	Blood pressure declined suddenly during patient transfer (mediastinal flutter after pneumonectomy)	Ephedrine and dopamine administration
Tooth loss	3	Tooth fell into the trachea or mouth	Removal of the tooth
Cardiac arrest	1	Ventricular fibrillation occurred in the post-anaesthesia unit (PACU)	Cardio pulmonary resuscitation (survival)
Drug mislabelling (sufentanil mislabelled as fentanyl)	1	Chest wall stiffness	Muscle relaxant administration
Puncture hematoma	3	Swelling in the neck or arm	Pressure

The 83 cases of adverse events caused by S (Table 4) were classified into the following three categories: (1) cardiac arrest (43 cases) caused by the stimulation of the surgical procedure, of which 90% of the cases occurred during left thoracotomy (30 cases), but all cases recovered successfully; (2) massivehaemorrhage (33 cases) was the most common event, with 28 cases occurring during surgery, and 5 cases occurring during the postoperative recovery from anaesthesia, and 9 patients death (accounting for 75% of the mortality cases), and (3) other problems (7 cases), including damage to the contralateral pleura caused by intraoperative contralateral tension of the pneumothorax (6 cases) and 1 case of death from the accidental falling of a tumour thrombus from the left atrium during left upper lung cancer surgery.

**Table 4:** Details of surgery-related events

Categories	n	Clinical manifestation	Treatment	Outcome
Cardiac arrest	43	32 ventricular fibrillations and 11 ventricular stand-stills on ECG	Cardiopulmonary resuscitation	Survival
Massive haemorrhage	33	Haemorrhagic shock 27 in the operating room 6 in the PACU	Exploratory thoracotomy and bleeding cessation Anti-shock treatment	9 deaths 24 survivals
Tension pneumothorax	6	Contralateral tension pneumothorax	Closed thoracic drainage	Survival
Falling tumour thrombus	1	Atrium episporium tumour thrombus falling off	Cardiopulmonary resuscitation	Death

Cardiac arrests were diagnosed by: (1)ECG, ventricular fibrillation or ventricular stand-still; and (2) invasive arterial blood pressure, no IBP wave

Sixteen cases were caused by P&S&A (Table 5), including the following (1) Cardiac arrest (6 cases): three cases were ventricular fibrillation but were successfully resuscitated; the CPR times were 10 min, 39 min and 7 min, and 1 case was recovered under cardiopulmonary bypass. Two of the 3 cases were discharged, and 1 case died due to a recurrence of cardiac arrest 3 days after surgery. (2) Airway problems (5 cases): severe hypoxemia occurred during a tracheotomy under topical anaesthesia for a patient with tracheal stenosis after thyroid cancer chemotherapy. Another patient suffered from a membranous tracheal injury 24 cm from the incisors and mediastinal emphysema during an oesophagectomy. After oesophageal surgery, we performed a repeat thoracotomy. (3) Other cases, including 3 cases with unexpected re-expansion pulmonary oedema and 2 cases with unexpected arrhythmia (Table 5).

**Table 5:** Details of P&S&A Events

Categories	n	Clinical manifestation	Treatment	Outcome
Cardiac arrest	6	Cardiac arrest secondary to operation-induced arrhythmia, pulmonary infarction or mediastinal shift	CPR	1 death 5 survivals
Hypoxemia	3	Dyspnoea and convulsions during tracheotomy for a patient with tracheal stenosis after thyroid cancer chemotherapy	Emergency cardiopulmonary bypass	Survival
Worsening arrhythmia	2	Bradycardia and AVB I progressing to AVB III upon entering the operating room	Operation cancelled Pacemaker implanted	Survival
Re-expansion of pulmonary oedema	3	Onset of right-side pulmonary oedema in the PACU after VATS of the right pneumothorax, chest X-ray confirmed	Re-intubation with a double-lumen tube and ventilation with PEEP for 1 day	Survival
Airway injury	2	Membranous tracheal injury 24 cm from the incisors and mediastinal emphysema during an oesophagectomy	Tracheal tube maintained at 26 cm from the incisors; extubation 5 days later	Survival

*AVB* atrioventricular block, *PACU* post-anaesthesia care unit, *VATS* video-assisted thoracic surgery

## Discussion

Using a large clinical database at a single institution, we found a perioperative adverse events incidence of 0.2% for thoracic surgery, 0.7% for tracheal surgery, 0.3% for mediastinal surgery, and 0.4% for oesophageal surgery. This retrospective study revealed a relatively low incidence of adverse events following thoracic surgery that was comparable to several previously reported rates [15].

Perioperative adverse events include unfavourable intraoperative incidents and postoperative complications [11]. An “anaesthesia-related event” is defined as a death or serious injury and disability caused by anaesthesia or the actions of an anaesthesiologist. The use of anaesthesia is a significant predictor of “any severe outcome, including death.” [7] A preoperative visit to an anaesthesia medicine clinic can reduce day-of-surgery case cancellations and/or case delays [16]. Because many factors, such as anaesthesia, the operation, and changes in a patient’s condition, are difficult to identify during surgery, we focus on the period from the preoperative anaesthesia visit to 48 h after anaesthesia at our centre. We record any reason for unexpected events during this period (i.e., previously unexpected occurrence of intraoperative life-threatening events) for discussion and analysis to increase our experience and improve our work. The data revealed that the rate of preoperative adverse events in thoracic surgery was 0.2%. Although these serious adverse events are rare, the unexpected events are unpredictable, and once the risk occurs, the mortality rate is as high as 8%. Therefore, it is necessary to prepare all types of measures for emergency rescue before anaesthesia. Broadening the anaesthesiologist’s scope of practice via the Perioperative Surgical Home may promote standardization, improve clinical outcomes and decrease resource utilization by providing greater patient-centred continuity of care throughout the preoperative, intraoperative, and postoperative periods [17].

Only 16 adverse events (11%) in this group were caused by “P”. These events included 4 serious drug-related events, including 1 blood transfusion event (no mild to moderate responders) according to the anaesthesiologists’ judgement, and 7 rare sporadic events (Table 2), including 4 cases of severe hypoglycaemia-induced arrhythmia, which is extremely rare but can be confirmed with blood glucose monitoring and is easily addressed. Preoperative adverse events caused by “P” are difficult to predict, so it is important for anaesthesiologist to focus on rational, standardized and streamlined administration and the timely identification and treatment of adverse events [18]. A strong relationship between patient data and preoperative clinical decision-making could potentially be used to support clinical decision-making for preoperative management [19].

Up to 55% of all the identified adverse events were completely or partly caused by “S”, indicating that the surgical procedure was an important factor in perioperative adverse events. Nine of the 12 deaths in this study were directly related to “S” (75% of total mortality), including 1 case of a left atrial tumour thrombus falling and 8 cases of massive haemorrhage. Massive haemorrhage remains an uncontrollable risk in thoracic surgery. Whether a massive haemorrhage can be controlled depends not only on the surgeon but also on his/her knowledge of surgical indications. Surgeons should be careful during recurrent giant intrathoracic tumor surgery. In addition, it is necessary to avoid the misdiagnosis of aneurysms and falling tumour thrombi during surgery.

Among the adverse events, 35 were caused by “A” alone and 16 were caused by “P&S&A”, including 13 cases related to respiratory management (10 cases of unexpected difficult airway and 3 cases of airway problems after extubation during the recovery period). There were no adverse consequences after rescue, but the adverse events were more or less related to the anaesthesiologists’ overconfidence or lack of experience. For example, 10 cases of unexpected difficult airway could have been avoided with sufficient preoperative assessment and preparation. Overall, respiratory management, especially airway management, should receive considerable attention from the anaesthesiologist. When a patient is on the borderline between a normal and a difficult airway, the situation should be treated as a difficult airway.

Compared with other chest diseases, tracheal disease is rare and easy to misdiagnose. Before surgery, both the surgeon and the anaesthesiologist focus on control of the respiratory tract. Nevertheless, in this group, two patients developed critical conditions. The preoperative adverse events rate for the tracheal surgery patients reached 0.7%, which was significantly higher than the rates of the pulmonary, mediastinal and oesophageal surgery groups. Although emergency cardiopulmonary bypass rescued 2 patients, tracheal disease preoperative assessments are still a challenge. Therefore, it is necessary to be prepared to provide timely cardiopulmonary bypass for patients with airway challenges.

In this study, cardiac arrest was the major adverse event (0.08%, 50/62,571). The rate found in this study was higher than the rates of anaesthesia-related cardiac arrest reported in Japan, Canada and France(from 0.8 to 3.3 per 10,000) [4]. This difference likely occurred because our group comprised thoracic surgery patients. Large doses of opioids or thoracic epidural block were used to suppress the double-lumen endotracheal tube and included strong surgical stimulation, which could increase vagus nerve tension. Under such conditions, if the patient is stimulated by an external operation, arrhythmia is induced by the further imbalance of the autonomic nervous system. Of the 50 cases of cardiac arrest during surgery in our group, the patients had no symptoms, and awareness of the arrest often occurred when the electrosurgical interference electrocardiogram (ECG) failed to show a waveform. The surgeon could have been notified immediately by the rapid drop in blood pressure if invasive arterial pressure monitoring had been used. The patients underwent continuous cardiac massage (external or internal thoracic) after the surgeon’s rapid reading of the ECG, and drugs or electrical cardioversion were used according to the ECG type. All 50 of the cardiac arrest cases recovered within 3 min, and their anaesthesia recovery processes were the same as those of the other patients. All cases requiring resuscitation were caused by P&S&A rather than surgical factors alone; Providing continuous cardiac massage and an assessment of its effectiveness using invasive arterial blood pressure are important as a guide for medical intervention. Our data show that preoperative cardiac arrest is difficult to predict in thoracic surgery and can not be completely avoided; however, 43 of the 50 cases of cardiac arrest caused by S occurred during left thoracotomy, including 25 cases of lung surgery, and most cases occurred during hilar stretching or lymph node dissection. Eighteen cases were characterized by sinus arrest or pulseless electrical activity. The safe limits of tolerance in patients with sinus asystole or pulseless electrical activity are not clear; Therefore, it is necessary to adopt active treatment to prevent adverse consequences to the central nervous system. Ten cases were treated with atropine and the other 8 cases with adrenaline, but whether this event occurred because the left vagus nerve was close to the left pulmonary artery was unclear. These observations suggest that patients should be closely monitored before effective preventive measures are taken; In particular, the invasive arterial pressure sound alarms should be used to quickly detect cardiac arrest via ECG interference. Electrical cardioversion should be used when ventricular fibrillation occurs, and defibrillators should be used as conventional equipment in thoracic surgery.

There are some notable limitations of this study. First, the analyses were based on administrative coding data, which may be subject to reporting bias or coding errors. Second, the analysis was not limited to the ASA score, BMI, age and gender [20] at the greatest risk of preoperative adverse events during thoracic surgery. Third, events were categorized as occurring due to the patient’s pathogenic condition, surgical factors, anaesthesia factors and the interaction of these three factors based on our experience, which was a relatively subjective process.

In summary, surgical factors (primarily cardiac arrest and massive haemorrhage) are the leading causes of preoperative adverse events. In cases of cardiac arrest, timely detection and proper treatment can prevent adverse consequences. In contrast massive haemorrhage is a significant cause of mortality that can be prevented with the surgeon’s early diagnosis and suitable intervention. It is difficult to predict possible events related to patients’ pathogenic conditions; thus, such events must be identified and treated immediately. An important aspect of anaesthesia-related adverse events is respiratory management, indicating that this factor should be a focus during evaluation and that we should not be overconfident in our ability to control it. Incident reporting can be a powerful tool for developing and maintaining an awareness of risks in healthcare practice. Using incident reports to improve care is challenging, and this study highlights the complexities involved and the difficulties that staff face in learning from incident data [10, 21–23]. However, information from such reports may assist future evidence-based perioperative planning and the allocation of patients to high-acuity facilities.

## Conclusions

The rate of perioperative adverse events after thoracic surgery was 0.2%. Adverse events must be identified and treated immediately. An important factor in anaesthesia-related events was respiratory management. Two major clinical manifestations of surgery-related events were cardiac arrest and massive haemorrhage. Cardiac arrest was the major factor contributing to adverse events, but its adverse consequences could be avoided with timely discovery and proper treatment. Massive haemorrhage is a significant cause of mortality that can be prevented with a surgeon’s early diagnosis and appropriate interventions.

## Abbreviations

AEs: Adverse events
AIMS: Anaesthesia Information Management System
AVB: Atrioventricular block
CPB: Cardiopulmonary bypass
CPR: Cardio-pulmonary resuscitation
ECG: Electrocardiography
IBP: Invasive blood pressure
ICU: Intensive care unit
PACU: Post-anaesthetic care unit
PEEP: Positive end expiratory pressure
SCA: Sudden cardiac arrest
VATS: Video-assisted thoracic surgery
